# Preparation of Biocidal Nanocomposites in X-ray Irradiated Interpolyelectolyte Complexes of Polyacrylic Acid and Polyethylenimine with Ag-Ions

**DOI:** 10.3390/polym14204417

**Published:** 2022-10-19

**Authors:** Kristina V. Mkrtchyan, Vladislava A. Pigareva, Elena A. Zezina, Oksana A. Kuznetsova, Anastasia A. Semenova, Yuliya K. Yushina, Etery R. Tolordava, Maria A. Grudistova, Andrey V. Sybachin, Dmitry I. Klimov, Sergey S. Abramchuk, Alexander A. Yaroslavov, Alexey A. Zezin

**Affiliations:** 1 Enikolopov Institute of Synthetic Polymeric Materials, Russian Academy of Sciences, Profsoyuznaya St. 70, 117393 Moscow, Russia; 2 Department of Chemistry, M.V. Lomonosov Moscow State University, Leninskie Gory 1-3, 119991 Moscow, Russia; 3 Gorbatov Federal Research Centre for Food Systems, Talalikhina St. 26, 109316 Moscow, Russia; 4 Nesmeyanov Institute of Organoelement Compounds, Russian Academy of Sciences, Vavilova St. 28, 119334 Moscow, Russia

**Keywords:** polyacrylic acid, polyethyleneimine, interpolyelectrolyte complexes, metal–polymer nanocomposites, radiation-induced reduction, silver nanoparticles, biocidal activity

## Abstract

Due to the presence of cationic units interpolyelectrolyte complexes (IPECs) can be used as a universal basis for preparation of biocidal coatings on different surfaces. Metallopolymer nanocomposites were successfully synthesized in irradiated solutions of polyacrylic acid (PAA) and polyethylenimine (PEI), and dispersions of non-stoichiometric IPECs of PAA–PEI containing silver ions. The data from turbidimetric titration and dynamic light scattering showed that pH 6 is the optimal value for obtaining IPECs. Metal polymer complexes based on IPEC with a PAA/PEI ratio equal to 3/1 and 1/3 were selected for synthesis of nanocomposites due to their aggregative stability. Studies using methods of UV–VIS spectroscopy and TEM have demonstrated that the size and spatial organization of silver nanoparticles depend on the composition of polymer systems. The average sizes of nanoparticles are 5 nm and 20 nm for complexes with a molar ratio of PAA/PEI units equal to 3/1 and 1/3, respectively. The synthesized nanocomposites were applied to the glass surface and exhibited high antibacterial activity against both gram-positive (*Staphylococcus aureus*) and gram-negative bacteria (*Salmonella*). It is shown that IPEC-Ag coatings demonstrate significantly more pronounced biocidal activity not only in comparison with macromolecular complexes of PAA–PEI, but also coatings of PEI and PEI based nanocomposites.

## 1. Introduction

The composites containing silver nanoparticles are a prospective base for design of optical, catalytic, diagnostic or sensor devices [1,2,3,4,5,6,7]. However, the greatest efforts were focused on the production of biocidal materials with prolonged effect, because application of metal–polymer nanocomposites did not lead to bacterial adaptation effects, in contrast to antibiotics [6,7,8,9,10,11,12,13]. Metal polymer nanocomposites are able to release biologically active substances into the environment in a controlled and gradual manner [11,12,13,14,15,16,17,18,19]. Reduction of metal ions in aqueous solutions and polymer dispersions is a general method for producing nanocomposites. Functional groups of polyelectrolytes can effectively bind metal ions, and in addition, they are powerful stabilizers of metal nanostructures. Interpolymer and interpolyelectrolyte complexes have been widely used in recent decades as scaffolds for the synthesis of nanocomposites with controlled sizes and different spatial organization of nanoparticles [2,20,21,22,23,24,25]. The use of macromolecular complexes provides more efficient synthesis of nanocomposites, better stabilization conditions and effective size control due to the wide possibilities of tuning the interactions of diverse units with metal ions/nanoparticles. 

Nowadays much attention is paid to combat against microbiological contamination of surfaces, since bacterial and fungal infections are an important problem for medicine, the food industry and other areas of management. One of the most promising approaches to the production of biocidal films/coatings is the synthesis of the polymers with cationic groups [17,26,27,28]. Cationic polymers bind to the negatively charged surface of the cell and initiate the processes [29] that lead to its death. Dispersions of non-stoichiometric interpolyelectrolyte complexes (IPECs) are a flexible basis for obtaining coatings. Soluble IPECs may have an excessive content of positively and negatively charged groups, which ensures interaction with charged surfaces of various types. The mutual neutralization of the charges of the cationic and anionic polymer in the IPEC results in the formation of hydrophobic regions, which also leads to its binding to hydrophobic surfaces. In this way, macromolecular complexes can be fine-tuned to the properties of various surfaces. The use of water as a solvent has obvious advantages in terms of accessibility and environmental safety.

IPECs of polyacrylic acid (PAA) and polyethylenimine (PEI) capable of binding large amounts of metal ions (up to 50 wt.%) [20,21], due to the formation of strong triple complexes with carboxylate and nitrogen-containing units. The films and coatings of triple metal–polymer complexes were used for efficient preparation of biocidal composites with silver nanoparticles [2,30,31]. The radiation induced approach is especially promising due to the possibility of effective preparation of metal polymer nanocomposites without contaminants [1,15,32,33]. Among various potential applications of nanocomposites, we may highlight the preparation of antimicrobial films and coatings based on the insoluble films and coatings of stoichiometric complexes PAA–PEI loaded by silver nanoparticles [2,30,31,32]. It is important to note that the matrix of PAA–PEI complexes is expected to be stable under irradiation to moderate absorbed doses used for both sterilization and synthesis of nanocomposites [34]. 

The aim of the work was to obtain biocidal nanocomposites in soluble non–stoichiometric IPECs with PAA/PEI ratios equal to 1/3 and 3/1 for further obtaining coatings due to their aggregative stability. Complexes with both an excess of anionic and cationic groups have been used because they promise the potential adjustment of adhesive interactions to surfaces of various types and have different effects on the generation of metal nanostructures. This paper discusses the features of radiation-initiated formation of silver nanoparticles and the biocidal properties of applied coatings from IPECs and nanocomposites. Silver nanoparticles were obtained for the first time in irradiated dispersions of PAA–PEI complexes. The use of the radiation–chemical approach made it possible to correctly compare the formation processes and sizes of nanoparticles in complexes of various compositions and their components.

## 2. Materials and Methods

The following reagents were used to prepare the samples: polyacrylic acid (Mw = 100,000) from Sigma-Aldrich (St. Louis, MO, USA), polyethylenimine (Mw = 60,000) from Serva (Heidelberg, Germany) and silver nitrate of analytical grade from Reachim (Moscow, Russia). To obtain metal polymer complexes, a silver nitrate solution was added to polyelectrolyte solutions or IPEC dispersions in low light conditions, after which the values were adjusted to pH 6 using 0.1 M KOH or H_2_SO_4_ solutions-both from Reachim (Moscow, Russia). To study the formation of metal polymer nanocomposites, 0.1 wt.% nonstoichiometric complexes of the PAA and PEI units with a molar ratio 3/1 and 1/3 with concentration of 0.026 wt.% of AgNO_3_ moles of silver ions were used. 

Samples of metal polymer complexes were irradiated on an X-ray machine with a 5-BKhV-6W tube from Svetlana X-ray (St. Petersburg, Russia) with tungsten anode (applied voltage 45 kV, anode current 80 mA) in polymer test tubes of 5 mm diameter. The above conditions provided a uniform generation of radiolysis products. Irradiation was carried out in a water–alcohol mixture with an ethanol content of 10 vol.%. To prevent oxidation with oxygen dissolved in water, the irradiated solution was bubbled with argon of a special purity grade during the irradiation process. The dose rate was determined using a ferrosulfate dosimeter irradiated in the same geometry as the test sample. The dose rate was calculated taking into account the mass absorption coefficients and the effective energy of X-ray quanta [35], the value of which was 17 Gy/s.

The structure of the nanocomposite material was studied using a transmission microscope “Leo-912 AB OMEGA” with a resolution of 0.3 nm. Data on the size and spatial distribution of nanoparticles were obtained from analysis of about 150–300 objects in the TEM images. The UV-VIS spectra and turbidimetric data were measured by a spectrometer from Perkin Elmer Lambda 9 instruments with the optical range was 200–900 nm (Überlingen, Germany). For measuring turbidity, the portions of the guest polyelectrolyte (GPE) solutions were successively added to a solution of the host polyelectrolyte (HPE) at 1-min intervals between titrant additions. Measurements were performed under constant stirring at room temperature directly in a quartz cuvette. 

The objects of biocidal research were glass slides (76 mm × 26 mm) with polymer coatings applied from polyelectrolyte solutions or IPEC dispersions with concentration 0.1 wt.% using an airbrush. Daily suspensions were prepared from daily cultures of microorganisms of the genus *Salmonella* (amount was 2 × 10^6^ CFU) and *St. aureus* (amount was 2.4 × 10^6^ CFU). 100 µL of suspensions were applied on the slides with polymer coatings and samples were kept at 37 °C for 2 h. After the exposure, the glass was washed off with a swab soaked in saline solution. Washout screening was carried out on the surface of meat-peptone agar. The number of CFU was determined using a standard protocol [36]. Slide glasses without applied coatings were used as control samples. 

## 3. Results

The interaction of PEI with PAA was studied by means of turbidimetric titration. The addition of PEI to a 0.1 wt.% solution of PAA or PAA 0.1 wt.% solution of PEI leads to a gradual turbidity of the mixtures. 

**Figure 1:**
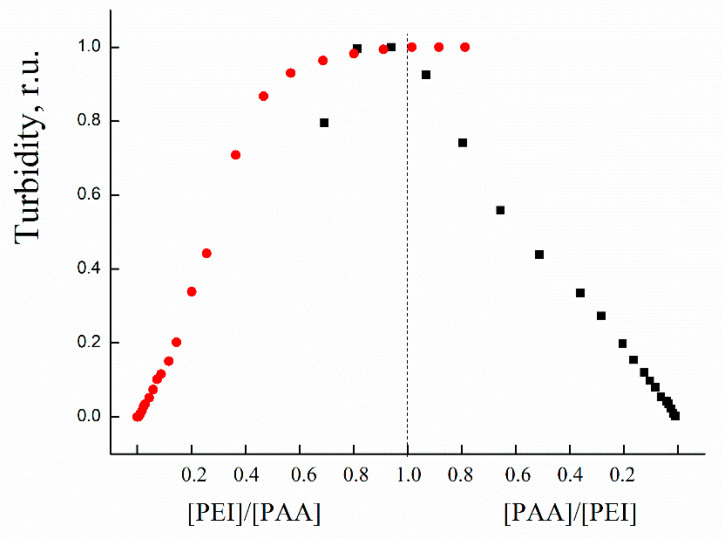
The dependence of relative turbidity at pH = 6 upon [PEI]/[PAA] ratio (curve 1 red) and [PAA]/[PEI] ratio (curve 2 black).

**Scheme 1:**
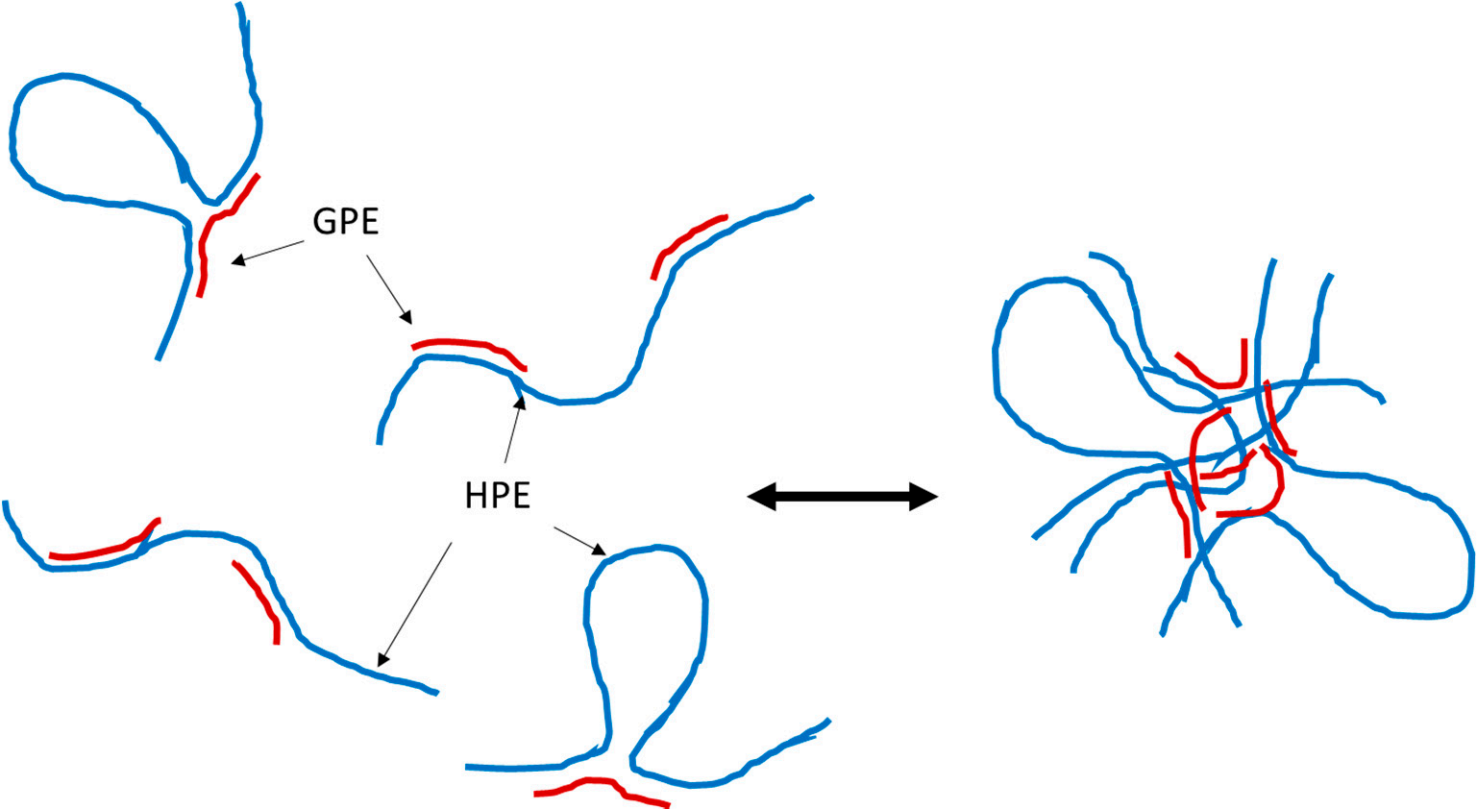
Non-stoichiometric interpolyelectrolyte complexes.

The results are presented in Figure 1 as dependence of relative turbidity upon the ratio of monomer units of polyacid and polybase. The addition of the solution of PAA to the solution of PEI in buffer with pH = 6, resulted in a progressive increase in the turbidity of the system reflecting formation of the IPEC and phase separation (see curve 1). This behavior is typical for mixtures of oppositely charged polyelectrolytes and indicates the formation of IPEC, which is insoluble in aqueous media, but can exist as colloidal particles with some excess of positively or negatively charged ionogenic groups. The polyelectrolyte that provides a molar excess of charged groups is named the “host” polyelectrolyte while the second component of IPEC is named the “guest”. The structure of the IPEC could be presented as a particle with several macromolecules included, so that areas of contact between guest and host polyelectrolytes form hydrophobic regions, excess units of host polyelectrolyte form (Scheme 1) charged loops and tails [37,38]. In turn, hydrophobic regions could contain areas of non-compensated charge of guest macromolecules due to non-complimentary location of cationic and anionic units and the presence of defects in polyelectrolytes. The structures of IPECs that could be formed upon mixing of the solutions of oppositely charged polyelectrolytes strongly depends on the mixing ratio, the consequence of adding the components to the mixture, degrees of polymerization of polymers and their ratio, ionic strength of the solution and so on [39,40,41]. As a result, structures with non-uniform distribution of charges, hydrophobic patches, and defect areas with non-compensated charged groups could be formed. As a result, these structures could serve as a good scaffold for the preparation of polymer–nanoparticle composites [42].

Maximum turbidity was reached at an equimolar ratio of monomer units of the polyelectrolytes. This proves that only electrostatic interactions take place between PEI and PAA at pH 6 i.e., all PEI units are protonated while all PAA units are dissociated, and no hydrogen bonds are formed. The effect of the additional charging of weak polyelectrolytes in the presence of the oppositely charged polyelectrolytes due to formation of the IPEC was described earlier [43]. In an excess of PEI, no water-soluble IPEC was obtained (see curve 2). The system with overall negative charge demonstrated phase separation at any polycation-to-polyanion ratio. The formation of non-soluble IPECs could be explained by the relatively high hydrophobicity of PEI, relatively high molecular weight dispersion of polymers and branched structure of PEI that could restrict distribution of polymer chains in IPECs [44]. 

Two IPECs with polyelectrolyte ratios [PAA]/[PEI] 3/1 and 1/3 were chosen to prepare ternary polyanion–(metal ion)–polycation complexes. The binary IPECs were characterized with DLS and laser microelectrophoresis with the following results: the IPEC with excess of polycation had a mean diameter of 200 nm with PDI 0.192, and an EPM value of 1.30 ± 0.11; the IPEC with an excess of polyanion had a mean diameter of 175 nm with PDI of 0.291 and an EPM value of −82 ± 0.17.

**Scheme 2:**
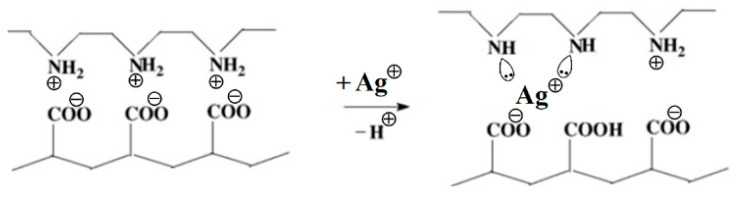
Formation of triple metal polymer complexes PAA–PEI-Ag^+^.

Studies of complexation in IPEC PAA–PEI have shown [21] that triple “sandwich” complexes PAA–(metal ions)–PEI are much stronger (Scheme 2) than the complexes of metal ions with functional groups of macromolecules PAA or PEI. The total content of polymer units in the IPEC dispersions was 1.8 × 10^−2^ M. To obtain metal polymer complexes, dispersions with a concentration of 1.5 × 10^−3^ M silver ions were used so that content of metal ions did not exceed the sorption capacity of the IPEC parts of non-stoichiometric complexes.

The preparation of metal polymer complexes as precursors for the synthesis of metal polymer nanocomposites was carried out at pH 6, because in acidic media the efficiency of the complexation of silver ions with functional groups is suppressed due to the competition of metal ion binding processes and protonation of functional groups of IPECs (Scheme 2). In alkaline media, precipitation of silver oxide occurs. The use of a pH value of 6 also ensures the effective formation of IPECs of PAA–PEI, so the protonation of nitrogen-containing units of PEI is 0.41 [21] and the degree of dissociation of carboxylate groups in this case is 0.44 [45].

**Figure 2:**
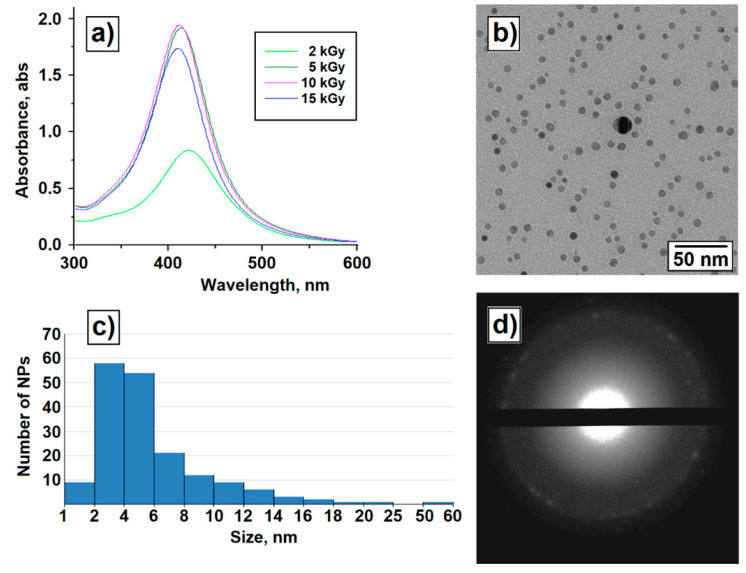
UV–visible spectra (**a**), micrography (**b**), histogram of the size distribution of nanoparticles (**c**), microdiffraction image (**d**) of irradiated PEI-Ag^+^ complexes.

**Figure 3:**
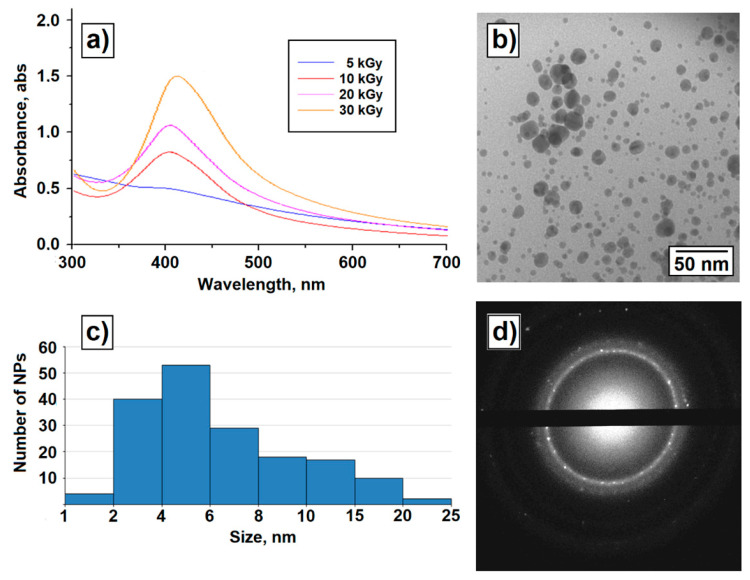
UV–visible spectra (**a**), micrography (**b**), histogram of the size distribution of nanoparticles (**c**), microdiffraction image (**d**) of irradiated PAA–PEI-Ag^+^ complexes, the molar ratio of PAA/PEI units equal to 1/3.

**Figure 4:**
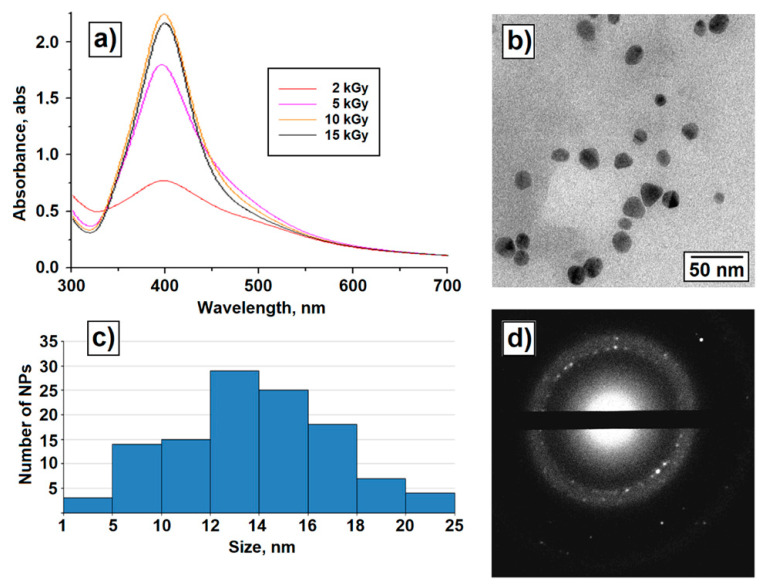
UV–visible spectra (**a**), micrography (**b**), histogram of the size distribution of nanoparticles (**c**), microdiffraction image (**d**) of irradiated PAA–Ag^+^ complexes.

**Figure 5:**
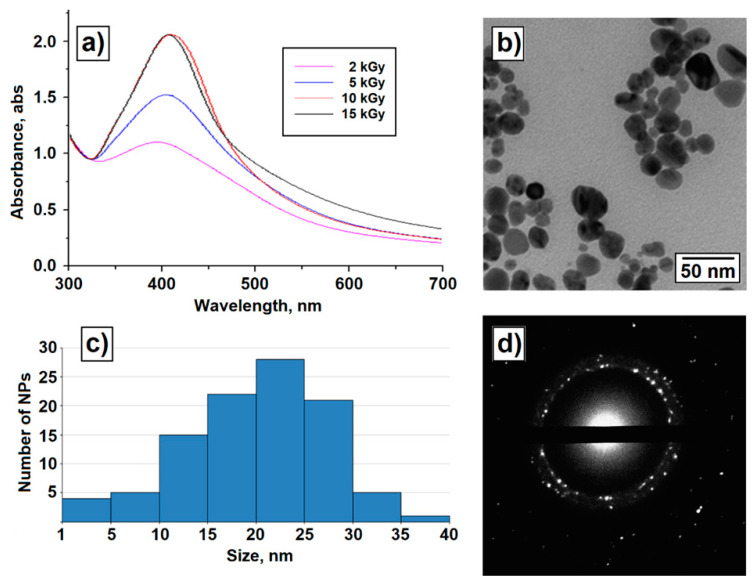
UV–visible spectra (**a**), micrography (**b**), histogram of the size distribution of nanoparticles (**c**), microdiffraction image (**d**) of irradiated PAA–PEI–Ag^+^ complexes with the molar ratio of PAA/PEI units equal to 3/1.

Irradiation of the samples with silver ions led to their coloring to yellow–gray. The formation of silver nanoparticles is proven by the fact that absorption bands with maxima from 400 nm to 414 nm (Figure 2, Figure 3, Figure 4 and Figure 5) are present on the optical spectra of all irradiated samples. An increase in the radiation dose leads to growth in the intensity of the absorption bands, which cease to increase after irradiation in a dose range of 15–30 kGy. To characterize nanoparticles on an electron microscope, samples irradiated to a dose of 15 or 30 kGy were used, which ensured the completion of the formation of nanoparticles in all irradiated systems. The reflections corresponding to interplane distances are observed on micro diffractograms: 2.35; 2.04; 1.45; 1.23 Å (Figure 2, Figure 3, Figure 4 and Figure 5), which corresponds to the values for the silver crystal lattice [46]. Thus, the analysis of diffraction data confirms that radiation-induced reduction of silver ions leads to the formation of metal nanoparticles.

With a minimum size, these particles are formed in irradiated solutions of polyethylenimine and complexes with an excess of polyethylenimine. The sizes of nanoparticles of particles are in the range 1–14 nm with a maximum distribution at 3 nm and in the range 1–24 nm with a maximum distribution at 5 nm, obtained, respectively, in irradiated PEI solutions and complexes with a molar ratio of PAA/PEI units equal to 1/3 (Figure 2 and Figure 3). Average sizes of nanoparticles synthesized in PAA solutions and complexes with a molar ratio of PAA/PEI units equal to 3/1 were 12 and 20 nm, respectively (Figure 4 and Figure 5), and the size distribution becomes broader. 

**Figure 6:**
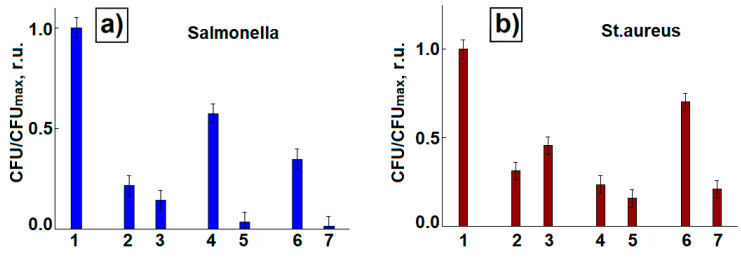
Biocidal activity of polymer coatings and nanocomposite coatings towards *Salmonella* (**a**) and *St. Aureus* (**b**): control (1); PEI (2); PEI/Ag (3); PAA/PEI 1/3 (4); PAA/PEI 1/3 + Ag (5); PAA/PEI 3/1 (6); PAA/PEI 3/1 +Ag (7).

Studies of the biocidal properties of coatings were carried out using both gram-negative and gram-positive bacteria. The results of the study of antibacterial action of the polymer-based coatings on glass are presented in Figure 6. For the species of *Salmonella* the coating of PEI demonstrated an efficiency to suppress bacterial growth—only 1/5 of the number of CFUs were detected on the coating in comparison to controls. Formation of the coatings from both negatively charged and positively charged IPECs resulted in a partial loss of the biocidal activity of PEI, however, the pronounced suppression of the bacterial growth was detected. Formation of coatings from the PEI/Ag nanocomposite resulted in a slight increase in biocidal activity in relation to PEI. The most effective antibacterial action was observed for the ternary nanocomposites, IPEC/Ag. Coatings from both negatively charged and positively charged nanocomposites allowed suppression of bacterial growth almost completely.

For the species of *St. Aureus*, the coating of PEI also demonstrated efficiency to suppress bacterial growth—only 1/3 of CFUs were detected on the coating in comparison to control. The coating from IPEC with PAA in excess demonstrated lower efficiency towards *St. Aureus* but the antibacterial properties of this coating were preserved. In contrast to *Salmonella* species, the coating from the PEI/Ag complex demonstrated a slight decrease in biocidal activity in comparison to the PEI coating. The coatings from the ternary IPEC/Ag nanocomposites demonstrated the most effective biocide activity towards *St. Aureus*—the suppression of the bacterial growth down to 15–20% was detected.

## 4. Discussion

For radiation-induced synthesis of nanoparticles in solutions and dispersions with 0.1 wt.% of polymers, the proportion of water is the overwhelming content. Due to this, the main role in the processes of reduction of metal ions and the formation of nanoparticles is played by processes involving products formed during radiolysis of water:

H_2_O → e_aq_^−^, **˙**OH, H_3_O^+^, H**˙**, H_2_, H_2_O_2_, HO_2_**˙** (1)

The main products of radiolysis of water are hydrated electrons, which have extremely high reduction potentials of −2.9 V [47] and such strong oxidizers as OH-radicals. To neutralize OH radicals and create favorable conditions for the reduction of metal ions, an additive of ethyl alcohol was used, while the radical CH_3_**˙**CHOH (reaction (2)) is formed, which has reducing properties (E_0_(CH_3_**˙**CHOH) = −1.4 V) [48]:

CH_3_CH_2_OH + **˙**OH → CH_3_**˙**CHOH + H_2_O (2)

Oxidation of CH_3_**˙**CHOH leads to the formation of a weak reducing agent-acetaldehyde [32]. Thus, when ethyl alcohol is used as a scavenger of OH radicals, only reducing particles are generated in water–organic mixtures.

The mechanisms of radiation-chemical formation of silver nanoparticles have been studied in detail [49,50,51]. In the first stage, silver ions are reduced to isolated atoms and clusters are formed:

(Ag)^+^ + e^−^_aq_ → (Ag)^0^ (3)

(Ag)^0^ + (Ag)^+^ → (Ag_2_)^+^ → (Ag_m_)^n+^ (4)

The growth of clusters by the reduction of silver ions on their surface (reaction (5)) and coalescence processes (reaction (6)) lead to the formation and enlargement of nanoparticles.

(Ag_m_)^n+^ + (Ag)^+^ → (Ag_m+1_)^n+1^→ (Ag_m+1_)^n^ (5)

(Ag_m_)^n+^ + (Ag_k_)^g+^ → (Ag_m+k_)^(n+g)+^ (6)

The process of reducing silver ions to isolated atoms (reaction (3), E^0^_(Ag+/Ag0)_ is more negative than −1.8 V [52]), which limits nucleation, and can provide such strong reducing agents as hydrated electrons. Reduction of silver ions on the surface of nanoparticles, which ensure the growth of nanoparticles (reaction (5)), may occur due to reactions of reducing agents with lower reduction potentials, including acetaldehyde [32]. 

Analysis of the UV–visible spectra and microdiffraction data (Figure 2, Figure 3, Figure 4 and Figure 5) shows that irradiation leads to the formation of silver nanoparticles with metal lattice in all the studied samples. Electron microscopy data also show the spatial ordering of nanoparticles. Previously, similar hierarchical structures were observed in IPEC PAA–polyalliamine and PAA–polyvinylimidazole [25,53] due to the formation of micro- and nano-particles of IPEC complexes filled by metal nanoparticles. 

Experimental data show that the composition of polymer solutions and dispersions affects the size of the resulting nanoparticles and their spatial organization. In polymer systems based on polyelectrolytes, the formation of nanoparticles occurs under conditions of electrostatic interactions of charged functional groups and hydrophobic interactions of the main chain with the surface of nanoparticles. From the point of view of electrostatic interactions, polycations should provide the least favorable conditions for the stabilization of positively charged nanoparticles. At pH 6, the degree of protonation of PEI is about 0.41. Taking into account the fact that every second nitrogen-containing group is charged in fully protonated polyethylenimine, the fraction of charged groups in experimental conditions is about 20%. Nevertheless, polyethylenimine solutions have demonstrated the highest stabilizing ability. In this case, nanoparticles with an average size of 3 nm and the narrowest size distribution occur (Figure 2). In this situation, the stabilization of nanoparticles can be provided by hydrophobic interactions of hydrocarbon groups with the surface of nanoparticles. Nitrogen-containing groups are strong ligands for silver ions, therefore, another powerful stabilizing factor we can assume due to the interaction of a significant proportion of uncharged amino groups and silver ions adsorbed on the surface of nanoparticles due to incomplete reduction processes. Micrography analysis also reveals specifically elongated structures of 3–5 isolated nanoparticles. The observed phenomenon is caused by mutual repulsion of both positively charged nanoparticles immobilized in macromolecules and positively charged PEI units in which metal nanostructures are immobilized.

The results obtained, show that the electrostatic interactions of PAA polyanions with the oppositely charged surface of nanoparticles provide significantly less favorable conditions for their stabilization than hydrophobic interactions in the case of PEI. The average size of nanoparticles in this case is 12 nm (Figure 3). Micrographs of irradiated solutions also show a specific spatial organization of nanoparticles. The balance of repulsion of positively charged nanoparticles and negatively charged links and attraction of opposite charges leads to their close location in aggregates.

The formation of nanoparticles of nanometer scale also occurs in the irradiated dispersions of interpolyelectrolyte complexes with an excess of polyanionic or polycationic component (Figure 3 and Figure 5). However, in both cases, their sizes are larger than the sizes of nanoparticles formed in solutions of polyelectrolytes (Figure 2 and Figure 4). On TEM images of irradiated IPEC with an excess of polyethylenimine both relatively small nanoparticles (up to 6 nm in size) and large nanoparticles with a tendency to aggregation are clearly visible (Figure 3). The obtained results clearly show that in this case, the immobilization of nanostructures occurs mainly separately in PEI and PAA units. However, experimental results do not exhibit such separation of nanostructures in IPEC with PAA excess. In this case, aggregates of relatively large nanoparticles are observed also as for individual PAA macromolecules. Thus, analysis of micrographs does not reveal an effect of the polycationic component on sizes and spatial distribution of nanoparticles, unlike IPEC with an excess of PEI. Consequently, the presence of an excess of PAA units prevents the hydrophobic interactions of PEI directly with nanoparticles. Moreover, nanoparticles with an average size exceeding the average size of nanoparticles formed in PAA solutions are formed (Figure 4 and Figure 5). Thus, in this case, the presence of polycations also worsens the electrostatic stabilization conditions, possibly due to partial blocking of negatively charged carboxylate groups. 

Study of antibacterial activity have demonstrated that PEI coatings provide a multiple increase in biocidal activity for both *Salmonella* and *Staphylococcus aureus* compared to the control sample. Recent publications have demonstrated antimicrobial activity of branched polyelectrolytes like PEI and silver nanoparticles [54,55,56,57,58,59]. We focus on possible cumulative action of polycation and nanoparticles. In this case the presence of silver nanoparticles either slightly decreases the antibacterial activity during tests of *Staphylococcus aureus*, or in the case of gram-negative bacteria (*Salmonella*) there is a slight increase in antibacterial effect. A probable explanation for this effect may be the strong interaction of PEI with the surface of nanoparticles, which not only leads to the formation of relatively small nanoparticles, but also ensures mutual passivation of both biocidal components. With a decrease in the content of PEI in the IPEC samples, as a rule, a significant decrease in biocidal activity occurs. Such phenomenon is logically interpreted as a diminution in the content of the polycationic biocidal component. The presence of silver nanoparticles in IPEC coatings leads to a significant increase in their antibacterial activity. The effect is more pronounced in the case of biocidal activity for *Salmonella*. Therefore, in this case, the role of silver nanoparticles for the destruction of *Salmonella* is many times higher than for the effect against *Staphylococcus aureus* which correlates with the fact that gram-positive microorganisms have less sensitivity to silver colloids [60]. Deposition of IPEC on the surfaces, either hydrophilic or hydrophobic, results in formation of a coating that has high resistance to wash-off in comparison to cationic polyelectrolyte [61]. Thus, preparation of nanocomposites of IPECs with silver nanoparticles has great potential for the creation of effective biocidal coatings.

## 5. Conclusions

The results of the work reveal the possibility of effective radiation chemical production of silver nanoparticles, both in the studied solutions of polyelectrolytes and in non-stoichiometric IPEC PAA–PEI of various compositions. The PAA–PEI–silver coatings applied to glass slides retained their inherent yellow–gray color for 14 days before antibacterial tests, which shows the high stability of the obtained nanocomposites. PEI solutions demonstrated the highest ability to stabilize nanoparticles among studied polymers. However, the reverse side of the coin in this case, is the passivation of the antibacterial activity of silver nanoparticles. A decrease in the content of the cationic component in IPEC, as a rule, leads to a significant decrease in the biocidal activity of macromolecular complexes compared with coatings of individual PEI. However, IPEC coatings containing silver nanoparticles show multiple higher biocidal activity, not only in comparison with macromolecular complexes of PAA–PEI, but also PEI coatings. Thus, the results obtained show the prospects of using IPEC–Ag nanocomposites for the further development of biocidal coatings with adjustable adhesion to surfaces of various types.

## Data Availability

Not applicable.
